# Oral Manifestations in Patients with Inflammatory Bowel Disease: A Systematic Review and Meta-Analysis

**DOI:** 10.3390/dj14050250

**Published:** 2026-04-23

**Authors:** Veronica Scocca, Giovanni Sarnelli, Marcella Pesce, Carlos Navarro-Cuéllar, Giovanni Dell’Aversana Orabona

**Affiliations:** 1 Maxillofacial Surgery Unit, Department of Neurosciences, Reproductive and Odontostomatological Sciences, University Federico II of Naples, Via Pansini 5, 80131 Naples, Italy; giovanni.dellaversanaorabona@unina.it; 2 Department of Clinical Medicine and Surgery, University of Naples Federico II, Via Pansini 5, 80131 Naples, Italy; giovanni.sarnelli@unina.it (G.S.); marcella.pesce@unina.it (M.P.); 3 Maxillofacial Surgery Department, Hospital General Universitario Gregorio Marañón, 28007 Madrid, Spain; canava03@ucm.es

**Keywords:** inflammatory bowel disease, Crohn’s disease, ulcerative colitis, oral manifestations, oral lesions, extraintestinal manifestations, oral ulceration, xerostomia, halitosis, oral microbiome

## Abstract

**Background/Objectives:** Oral manifestations are recognized extra-intestinal features of inflammatory bowel disease (IBD); however, their prevalence and clinical relevance remain controversial. This study aims to quantify the prevalence of individual oral outcomes in IBD patients and to evaluate their association with the disease compared with healthy controls. **Methods:** A systematic review was conducted in accordance with PRISMA guidelines. Eligible studies were identified through searches of PubMed/MEDLINE, Cochrane Library, Scopus, Ovid MEDLINE, and EMBASE. Studies reporting oral signs and symptoms in IBD patients were included. A single-arm meta-analysis was performed for oral ulcerations, dry mouth, halitosis, tongue alterations, oral aphthae, stomatitis, and taste changes. Risk of bias was assessed using the Newcastle–Ottawa Scale. **Results:** Twenty-one studies including 7791 participants (5914 IBD patients and 1877 controls) were analyzed. The pooled prevalence of oral ulcerations was 20% (95%CI11–33), dry mouth 32% (95%CI14–59), halitosis 22% (95%CI7–51), and tongue alterations 11% (95%CI4–24). Comparative analyses showed no statistically significant differences between IBD patients and controls for these outcomes. **Conclusions:** Although oral manifestations are frequently reported in IBD patients, their prevalence does not significantly differ from that of the general population. Standardized, multicenter studies are required to clarify disease-specific associations.

## 1. Introduction

Inflammatory bowel disease (IBD) is a chronic, relapsing inflammatory disorder of the gastrointestinal tract, encompassing two main entities: ulcerative colitis (UC) and Crohn’s disease (CD) [1]. Although its precise etiopathogenesis remains unclear, IBD is widely recognized as a multifactorial condition involving a complex interplay among genetic susceptibility, environmental influences, dysbiosis of the gut microbiota, and dysregulated immune responses. This multifactorial nature reflects the broad spectrum of symptoms that IBD patients may experience beyond gastrointestinal issues, commonly referred as extra-intestinal manifestations (EIM). The prevalence of EIM varies widely, ranging from 16.7% to 40% [2,3], and may include arthritis, skin disorders, eye inflammation, liver problems, and oral cavity issues. Several oral manifestations have been described in patients with IBD, with a reported prevalence ranging from 5 to 50% [4,5,6]. Moreover, oral involvement does not necessarily coincide with active gastrointestinal disease [5,7] and may precede gastrointestinal symptoms by several months or even years [8,9]. Oral manifestations of IBD can be either specific or nonspecific, due to intestinal malabsorption or induced by pharmacological treatments.

Despite several reports describing oral involvement in IBD, the relationship between oral manifestations and disease status remains unclear. Therefore, this systematic review and meta-analysis aimed to provide a comprehensive quantitative assessment of their prevalence and potential association with IBD. The primary objective was to estimate the pooled prevalence of individual oral manifestations in IBD patients. A secondary exploratory objective was to compare selected outcomes with healthy controls when sufficient comparative data were available.

## 2. Materials and Methods

The study followed the Preferred Reporting Items for Systematic Reviews and Meta-Analyses (PRISMA) guidelines. Since it involved a review of previously published studies, neither ethics approval nor informed consent were required. Additionally, the review was registered in the PROSPERO database under the ID number 1186590. The completed PRISMA checklist [10] is provided as Supplementary Material File S1.

### 2.1. Search Strategy

The research covered the years 1950–2025 and included Pubmed/MEDLINE, Cochrane Library, Scopus, Ovid MEDLINE, and EMBASE databases. Relevant keywords, phrases and MeSH terms were tailored to meet the specific requirements of each individual database. An example of the search strategy was the one used for Pubmed/MEDLINE: (“Inflammatory Bowel Diseases”[Mesh] OR “Inflammatory Bowel Disease” OR “IBD” OR “Crohn Disease”[Mesh] OR “Crohn’s Disease” OR “Ulcerative Colitis”[Mesh] OR “Ulcerative Colitis”) AND (“Mouth Diseases”[Mesh] OR “Oral Manifestations” OR “Oral Lesions” OR “Oral Symptoms” OR “Oral Health” OR “Halitosis” OR “Taste Disorders” OR “Dry Mouth” OR “Stomatitis” OR “Aphthous Ulcers” OR “Oral Ulceration” OR “Tongue Disorders” OR “Mucosal Lesions”) AND (“Humans”[Mesh]). Then, a cross-reference search of the selected articles was conducted using the snowballing method to ensure the retrieval of all possible studies.

The last search was conducted on 21 October 2025.

### 2.2. Study Selection

This systematic review and meta-analysis was carried out according to the PICOTS acronym: Patients (P), patients with IBD diagnosis; Intervention (I), UC and CD; Comparison (C), IBD vs. healthy controls; Outcomes (O), Oral sign and symptoms; T (Timing), none; Study design (S), retrospective and prospective cohort studies, case–control and cross-sectional studies and randomized clinical trials (RCTs).

Studies were excluded if they were not available in full-text form; included <5 patients; the article type was either a case report, conference abstract, letter to the editor, or book chapter. Articles were also excluded if they did not report any oral signs or symptoms.

### 2.3. Data Collection Process

The search was conducted independently by two investigators (V.S. and G.D.A.O.). References from the identified databases were merged, and duplicates were removed using the reference management software EndNote^®^ 21 (version 21.5). Articles were screened for relevance based on title and abstract, with those deemed appropriately selected for full-text review. Any discrepancies between the screening authors were resolved through discussion until consensus was reached.

Systematic data extraction from the included studies was made using a structured form, with data archived in a customized Excel^®^ (Microsoft Corp, Seattle, WA, USA) spreadsheet. One author (V.S.) independently compiled a standardized form to extract the following characteristics of the included studies: authors, year of publication, study design, number of patients included in the study, mean age, number of patients with IBD diagnosis, number of healthy controls, oral sign and symptoms, DMFTe dmft Index, periodontal manifestations, pharmacological treatments, smoke habits. The accuracy of the extracted data was verified by another author (G.D.A.O.).

### 2.4. Data Synthesis and Analysis

All the articles included in the qualitative analysis were then included in the meta-analysis.

All clinical measures were reported as provided by the individual studies. When the mean follow-up time was not available, the median measure was used.

A single arm meta-analysis was performed for the rate of oral ulcerations, rate of dry mouth, halitosis, tongue alteration (included coated tongue, geographic tongue, fissured tongue), rate of oral aphtae, stomatitis and rate of taste changes. The results were presented as pooled estimates with 95% Cis, and a forest plot was generated for each outcome. To stabilize any variance in the analysis of proportions, the Freeman–Tukey double arcsine transformation was applied.

When at least four studies provided comparative data for both IBD patients and healthy controls, random-effects binary meta-analyses were performed using odds ratios (ORs) with 95% confidence intervals; *p*-values refer to the pooled comparative effect.

The Cochran’s Q test was applied to assess the degree of heterogeneity between the studies and I^2^ was calculated as a measure of heterogeneity. The I^2^ value represents the percentage of total variation between the studies caused by heterogeneity rather than by chance. According to the Cochrane criteria, values from 0% to 40% may represent low heterogeneity, 30% to 60% moderate heterogeneity, 50% to 90% substantial heterogeneity and 75% to 100% considerable heterogeneity.

A random-effects model was used for all meta-analyses, if the true effect size may vary across the studies due to differences in the study populations, methodologies or other sources of variability. This model accounts for both within-study and between-study heterogeneity, providing more conservative and generalizable effect estimates.

All the analyses were performed using the R software for statistical computing (R version 4.4.2; “meta” and “dmeta” packages).

### 2.5. Risk of Bias and Study Quality Assessment

Two authors (V.S. and G.D.A.O.) assessed the quality of each study using the Newcastle-Ottawa Quality Assessment Scale. A sensitivity analysis was conducted in this review when more than six studies were available for a given outcome, in order to assess the robustness of the pooled estimates and to explore the impact of potential sources of heterogeneity. Publication bias was assessed using funnel plots and Egger’s linear regression test when at least 4 studies were available, in accordance with the Cochrane guidelines.

## 3. Results

### 3.1. Study Selection

**Figure 1:**
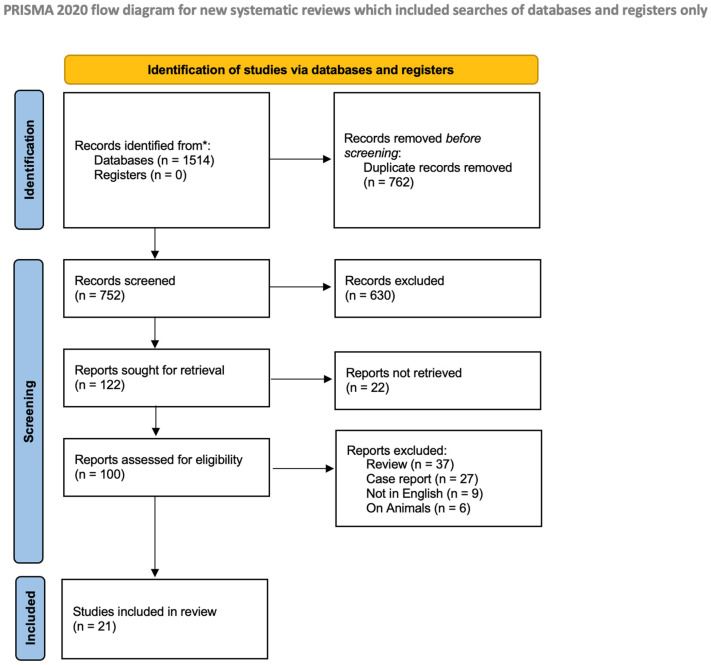
PRISMA flow diagram.

The study selection process is illustrated in Figure 1.

The initial search yielded 1514 records. Of these, 762 duplicates were removed before screening. After the initial screening of titles and abstracts, 630 articles were excluded as off-topic and 22 after abstract assessment, leaving 100 articles for full-text evaluation.

Among these, 79 studies were excluded after full-text assessment for the following reasons: review articles *(n* = 37), case reports (*n* = 27), non-English language (*n* = 9), and animal studies (*n* = 6). Ultimately, 21 studies met the inclusion criteria.

Ultimately, 21 publications met the inclusion criteria and were included in both the qualitative and quantitative (meta-analysis) synthesis.

### 3.2. Description of the Studies

The general characteristics of the included studies are summarized in Table 1. All studies were published in English. Twelve studies were case–control in design, while the remainder were observational (cohort and cross-sectionals). Eight studies were published in the 2000, nine in the 2010s, and four in the 2020s.

### 3.3. Study Results

A total of 7791 patients were included in the quantitative analyses, comprising both healthy controls and IBD patients. Overall, 53% were male (*n* = 2798/5291) with a mean age of 39.0 ± 40.2 years (95%CI 6–49). Among the 5914 patients diagnosed with IBD (76% of the cohort), 70% had Crohn’s Disease (*n* = 4065/5914) and 30% had Ulcerative Colitis (*n* = 1772/5914). In the IBD group, 20% of the patients were current smokers (*n* = 740/3790), a proportion comparable to that observed in the control group (*n* = 186/956).

The prevalence of smoking among IBD patients (20%, *n* = 740/3790) was similar to that of healthy controls (19%, *n* = 186/956), with no statistically significant difference (*p* = 0.29)

### 3.4. Oral Signs/Symptoms in IBD Patients

**Figure 2:**
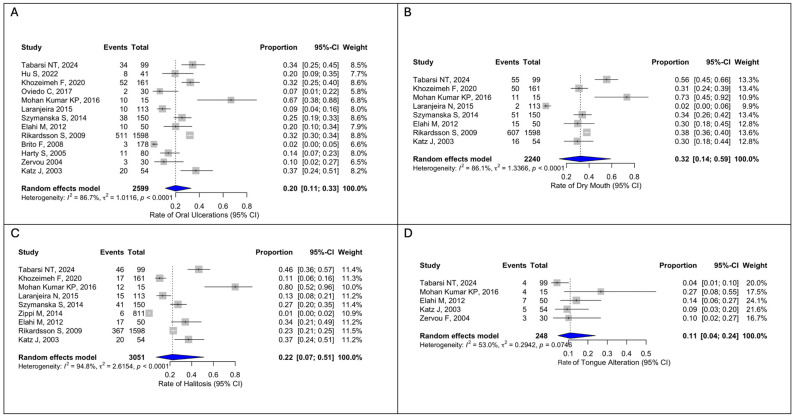
(**A**) Forest plot for oral ulcerations; (**B**) Forest plot for dry mouth; (**C**) Forest plot for halitosis; (**D**) Forest plot for tongue alterations. Tabarsi NT, 2024 [12]; Hu S, 2022 [13]; Khozeimeh F, 2020 [14]; Oviedo C, 2017 [15]; Mohan Kumar KP, 2016 [16]; Laranjeira N, 2015 [18]; Szymanska S, 2014 [19]; Zippi M, 2014 [3]; Elahi M, 2012 [20]; Rikardsson S, 2009 [22]; Brito F, 2008 [6]; Harty S, 2005 [24]; Zervou F, 2004 [26]; Katz J, 2003 [27].

The pooled prevalence of oral ulcerations in patients with IBD was 20% (722/2599; 95%CI 11–33), with moderate between-study heterogeneity (I^2^ = 86.7%, Q = 1.0116, *p* < 0.0001) (Figure 2A). Dry mouth was more frequent, with a pooled rate of 32% (822/2240; 95%CI 14–59), also showing moderate heterogeneity (I^2^ = 86.1%, Q = 1.3366, *p* < 0.0001) (Figure 2B). Halitosis was reported in 22% of patients (543/2938; 95% CI, 7–51), but with high heterogeneity (I^2^ = 94.8%, Q = 2.6154, *p* < 0.0001) (Figure 2C). Tongue alterations occurred in 11% of cases (30/248; 95%CI 4–24), with moderate heterogeneity (I^2^ = 53.0%, Q = 0.2942, *p* = 0.0746) (Figure 2D).

**Figure 3:**
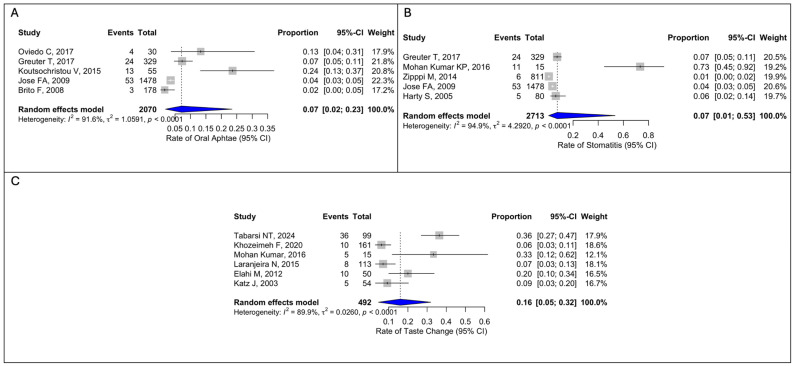
(**A**) Forest plot for oral aphtae; (**B**) Forest plot for stomatitis; (**C**) Forest plot for taste change. Oviedo C, 2017 [15]; Greuter T, 2017 [2]; Koutsochristou V, 2015 [17]; Jose FA, 2009 [9]; Brito F, 2008 [6]; Mohan Kumar KP, 2016 [16]; Zippi M, 2014 [3]; Harty S, 2005 [24]; Tabarsi NT, 2024 [12]; Khozeimeh F, 2020 [14]; Laranjeira N, 2015 [18]; Elahi M, 2012 [20]; Katz J, 2003 [27].

Oral aphthae and stomatitis were less common, each with a pooled prevalence of 7% (97/2070, 95%CI 2–23; 99/2713, 95%CI 1–53), showing high heterogeneity (Figure 3A,B). Taste changes were observed in 16% of patients (84/492; 95%CI, 5–32), also with high heterogeneity (I^2^ = 89.9%, Q = 0.0260, *p* < 0.0001) (Figure 3C).

### 3.5. IBD Patients vs. Healthy Controls

Twelve case–control studies compared oral manifestations in patients with IBD and healthy controls. Across all outcomes analyzed, no statistically significant differences were observed. Specifically, lip/gingival swelling (*p* = 0.650), taste changes (*p* = 0.618), burning sensation (*p* = 0.5498), dry mouth (*p* = 0.157), halitosis (*p* = 0.165), acidic taste (*p* = 0.805), oral ulcerations (*p* = 0.338), and tongue alterations (*p* = 0.911) did not differ significantly between the two groups.

### 3.6. Periodontal Manifestation

Out the 21 included studies, only seven reported about periodontal manifestation [1,6,16,22,23,24,27]. No quantitative analysis was conducted for periodontal manifestations, as the included studies employed heterogeneous outcome measures.

Two studies [6,15] reported significantly higher DMFT and dmft scores in IBD patients compared to controls (*p* < 0.001 and *p* = 0.018, respectively), suggesting greater caries experience. Conversely, other studies [23] did not find statistically significant differences. Several studies found higher periodontal index values, gingival inflammation, and probing depth in IBD patients [6,21,26]. Habashneh et al. [21] observed increased CAL and bleeding on probing in both CD and UC compared with controls. Similarly, Brito et al. [6] reported significantly higher PPD and CAL in both CD and UC groups (*p* < 0.001). Zervou et al. [26] found significantly greater gingival bleeding (*p* < 0.0001) and gingivitis (*p* = 0.002) in IBD patients. Rikardsson et al. [22] also reported higher rates of gingival bleeding (41%, *p* < 0.001) and periodontitis (7%, *p* = 0.028).

### 3.7. Risk of Bias Assessment

The methodological quality of the included studies was assessed using the Newcastle-Ottawa Scale), with scores ranging from 5 to 9 and a mean score of 7.2. (Table 2).

**Figure 4:**
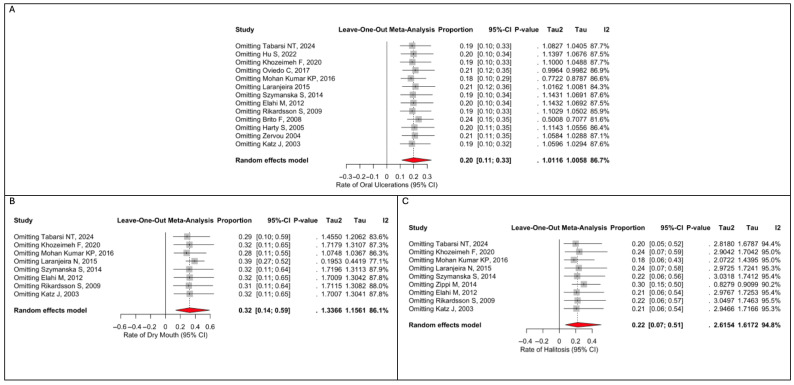
(**A**) Leave-one-out sensitivity analysis for pooled oral ulcerations rate; (**B**) Leave-one-out sensitivity analysis for pooled dry mouth rate; (**C**) Leave-one-out sensitivity analysis for pooled halitosis rate. Tabarsi NT, 2024 [12]; Hu S, 2022 [13]; Khozeimeh F, 2020 [14]; Oviedo C, 2017 [15]; Mohan Kumar KP, 2016 [16]; Laranjeira N, 2015 [18]; Szymanska S, 2014 [19]; Elahi M, 2012 [20]; Rikardsson S, 2009 [22]; Brito F, 2008 [6]; Harty S, 2005 [24]; Zervou F, 2004 [26]; Katz J, 2003 [27]; Zippi M, 2014 [3].

Leave-one-out sensitivity analysis confirmed that the pooled estimates for oral ulcerations, dry mouth, halitosis remained stable, with no single study exerting a disproportionate influence on the overall results. This indicates that the findings were robust and not driven by individual studies. (Figure 4).

**Figure 5:**
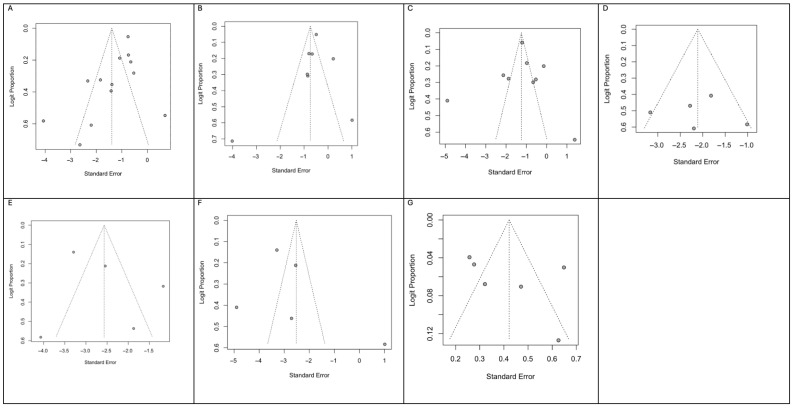
(**A**) Funnel plot for pooled oral ulcerations rate; (**B**) Funnel plot for pooled dry mouth rate; (**C**) Funnel plot for pooled halitosis rate; (**D**) Funnel plot for pooled tongue alterations rate; (**E**) Funnel plot for pooled oral aphthae rate; (**F**) Funnel plot for pooled stomatitis rate; (**G**) Funnel plot for pooled taste changes rate.

Funnel plots for each outcome are shown in Figure 5. Visual inspection and Egger’s linear regression test did not show evidence of small-study effects for oral ulcerations: z = –1.8772, *p* = 0.0605; dry mouth: z = –1.0352, *p* = 0.3006; halitosis: z = 0.2765, *p* = 0.7821; tongue alterations: z = 0.2378, *p* = 0.8121; oral aphthae: z = –0.0019, *p* = 0.9985; stomatitis: z = 0.8979, *p* = 0.3692; taste changes: z = 1.1403, *p* = 0.2542.

## 4. Discussion

The oral cavity is frequently affected in patients with IBD, particularly in those with CD, with a reported prevalence ranging from 5% to 50% [4,5,6]. This wide variation likely reflects the heterogeneity among studies and patient populations, influenced by factors such as sex, age, genetic background, ethnicity, and even differences in investigators’ diagnostic expertise, as observed in our qualitative and quantitative analysis. These inconsistencies highlight the need for a systematic and standardized evaluation of oral manifestations in IBD patients, and call for caution in the interpretation of these results. Importantly, many of the included studies did not report data stratified by CD and UC, limiting disease-specific analyses and potentially attenuating signals related to distinct pathophysiological mechanisms.

Rather than focusing solely on the overall prevalence of oral signs and symptoms in IBD, this study aimed to quantify individual oral outcomes to better assess their potential association with the disease. Instead of categorizing oral manifestations as specific or nonspecific, we opted for a data-driven approach, analyzing each clinical feature independently. This strategy allowed for a more objective interpretation of the available evidence and helped to minimize the bias introduced by the variability in diagnostic criteria and clinical expertise among studies. This approach was specifically chosen to avoid overinterpretation of prevalence data and to reduce the risk of inferring causal associations based solely on frequency estimates. Accordingly, the prevalence estimates reported herein should not be interpreted as evidence of disease specificity, but rather as an overview of the frequency with which these manifestations have been reported in IBD populations.

Dry mouth was the most frequently reported oral symptom among IBD patients, consistent with the findings of Tabarsi et al. [12], and is likely influenced by pharmacological treatments. Mohan Kumar et al. [16] observed the highest prevalence, with 11 out of 15 patients affected, despite the small sample size. They also measured salivary flow rate (ml/min) and found it decreased compared to controls (*p* = 0.02), which may be attributable to long-term use of steroids and sulfasalazine, both of which can reduce the secretory capacity of salivary acinar cells. However, in our pooled analysis, there was no statistically significant difference in the prevalence of dry mouth between IBD patients and healthy controls. Similarly, Tabarsi et al. [12] reported no statistically significant association between UC severity and dry mouth. Taken together, these findings suggest that xerostomia may be common in IBD patients but not necessarily disease-specific, as it may also reflect treatment-related and nonspecific systemic factors.

Elahi et al. [20] reported a statistically significant association between severe UC and oral manifestations, including tongue coating (*p* < 0.0001), halitosis (*p* < 0.0001), and oral ulceration (*p* = 0.001), compared to controls. These findings suggest a possible link between disease severity and oral symptoms, potentially influenced by medication use. However, Tabarsi et al. [12] did not observe any significant association between UC severity and dysphagia, nausea, halitosis, food or acid reflux, oral ulcers, geographic tongue, fissured tongue, or coated tongue, regardless of medication usage. This aligns with our findings, in which no statistically significant differences were observed for halitosis, tongue alterations, or oral ulcerations between IBD patients and healthy controls. Overall, the available evidence does not support a consistent association between these manifestations and IBD, although the limited number of comparative studies and substantial heterogeneity warrant cautious interpretation.

Oral ulcerations were the most reported oral sings, with a pooled rate of 20% (IC95% 11–33). Mohan Kumar et al. [16] reported 10 out of 15 UC patients suffering aphtous ulceration, likely related to micronutrient malabsorption deficiencies such as iron, leading to iron deficiency anemia, and vitamin B12, as seen in pernicious anaemia. These findings align with Correll et al. [28], who suggested that recurrent oral ulcers should prompt evaluation for potential systemic disease. Nevertheless, the high prevalence of oral ulcerations does not necessarily imply disease specificity, as nutritional deficiencies and systemic inflammation may act as indirect or confounding mechanisms. Moreover, recurrent aphthous stomatitis is also common in the general population, which may partly explain the lack of significant differences observed in comparative analyses.

Hu et al. [13] characterized oral microbiome of CD patients with/without oral manifestations, observing significant differences between subjects with and without CD, as well as amongst CD subjects with and without oral lesions. Four microbial species were found associated with oral ulcers (*Turicimonas muris*, *Anaerostipes hadrus*, *Clostridium bolteae* and *Roseburia inulinivorans*). Additionally, an increase in microbial enzymes associated with butyrate metabolism was positively associated with the presence of oral ulcers, specifically enriching the acetyl-CoA-based pathway for butyrate production. These findings are intriguing, but they should be interpreted as exploratory, particularly because microbiome composition may also vary according to age, diet, treatment exposure, and disease phenotype.

To the best of our knowledge, the most recent systematic review on the prevalence of oral lesions and their correlation with IBD dates back to 2019 [5]. The presence of oral manifestations and symptoms in IBD, along with the higher risk of caries and periodontitis, is a well-recognized concern among clinicians. However, as far as we know, no prior attempt has been made to quantitatively synthesize the current literature in order to provide a comprehensive estimate of prevalence and assess potential associations with the disease. In this regard, our study should be viewed primarily as a quantitative synthesis of prevalence data, with comparative analyses serving as secondary and exploratory assessments where sufficient data were available.

Our study has several limitations that must be acknowledged. The included studies were highly heterogeneous due to differences in diagnostic criteria, clinical assessment tools, and patient characteristics (age, sex, ethnicity, and disease severity). Age-stratified analyses were not feasible because most studies did not report outcomes in a sufficiently disaggregated manner. Additionally, most studies were observational, and some had small sample sizes, reducing the statistical power of the analyses. The lack of uniform definitions for oral manifestations represents a further source of bias. These limitations highlight the need for future standardized, multicenter studies with larger cohorts to clarify the true relationship between IBD and oral manifestations and to guide clinical practice. Furthermore, the frequent inclusion of patients labelled generically as “IBD,” without distinction between CD, UC, or IBD-unclassified, represents an intrinsic limitation that could not be resolved within the available data. The inclusion of both pediatric and adult populations likely introduced additional heterogeneity, particularly with respect to oral microbiome composition, dentition-related outcomes, medication exposure, and prevalence of oral lesions.

In summary, oral manifestations are relatively common manifestations in patients with IBD, with a wide variation in prevalence across studies. However, our results suggest that, despite their frequency, symptoms such as dry mouth, halitosis, tongue alterations, and oral ulcerations did not show consistent statistically significant differences between IBD patients and healthy controls. This indicates that many of these manifestations may be common in the general population and not necessarily specific to the disease. Evaluating individual symptoms rather than overall prevalence allows for a more precise assessment of their potential association with IBD. Future studies with standardized diagnostic criteria and stratification by disease subtype are needed to clarify disease-specific oral phenotypes. Therefore, the current evidence should be interpreted as insufficient to support a consistent association between several common oral manifestations and IBD, rather than as evidence of no association. Clinically, recognizing these oral manifestations is important for early diagnosis and timely management. When patients present to our attention, assessment of oral lesions should be integrated into the overall clinical evaluation. Management should include both symptomatic care and coordination with the gastroenterology team to optimize treatment of the underlying disease, emphasizing a multidisciplinary approach to ensure comprehensive patient care. Emerging evidence on the role of the oral microbiome also provides interesting insights into the pathogenic mechanisms underlying oral lesions in IBD patients.

## Figures and Tables

**Table 1:** Qualitative assessment of included studies. RSP = retrospective; PSP = prospective; CC = case–control; CS = cross sectional; UC = ulcerative colitis; CD = Chron’s disease; BOP = presence of bleeding on probing; PPD = probing pocket depth; CAL = clinical attachment loss; RS = resective intestinal surgery; PSV = pyostomatis vegetans; Ctrl = control; * = median.

Author, Year	Study Design	Country	N (M/F)	IBD (M/F)	Ctrl (M/F)	Mean Age ± DS (Range)	Oral Sign and Symptoms in IBD and Ctrl Patients	DMFTe Dmft Index	Periodontal Manifestations	Pharmacological Treatments	Smoke Habits
Cagir Y, 2024 [11]	RSP	Switzerland	676 (404/272)	CD = 676 (404/272)	N/A	38 * (11–65)	cobblestoning = 2 orofacial granulomatosis = 3 glossitis (with fissuring) = 3 lip swelling (with fissuring) = 2	N/A	N/A	Mesalazine, sulfasalazine, budesonide, steroids, immuno-modulators	Former smoker = 255 Ex smokers = 168 (3; *p* = 0.333)
Tabarsi NT, 2024 [12]	CC	Iran	198 (100/98)	UC = 99 (50/49)	99 (50/49)	UC: 41.84 ± 11.66 Ctrl: 40.43 ± 12.67	taste change = 36 (*p* = 0.002), burning sensation = 12 (*p* = 0.037), dry mouth = 55 (0.171), halitosis = 46 (*p* = 0.28), acidic taste = 17 (*p* < 0.001), oral ulceration = 34 (*p* = 0.029), geographic tongue = 4 (*p* = 0.116), fissured tongue = 59 (*p* = 0.277), coated tongue *p* = 61 (*p* = 0.003)	N/A	N/A	N/A	N/A
Hu S, 2022 [13]	CS	China	65 (34/31)	CD = 41 (24/17)	24 (10/14)	39.5 (21–67) CD: 38.6 ± 12 Ctrl: 40.2 ± 1	16 oral ulcerations: 8, cobblestoning: 8	N/A	N/A	Azathioprine, steroids	Former/ex-smoker CD = 8, Ctrl = 4 Nonsmoker CD = 33, Ctrl = 20 (*p* = 0.913)
Khozeimeh F, 2020 [14]	CS	Iran	161 (87/74)	CD = 42 UC = 119	N/A	42 ± 11.71	CD: 17, UC: 35 Dry mouth = 50, taste changes = 10, acidic taste sensation = 10, halitosis = 17, oral ulcerations = 52	N/A	N/A	Mesalazine, sulfasalazine, azathioprine, steroids	Smoker = 22.2% Nonsmoker = 32.9% (*p* > 0.05)
Oviedo C, 2017 [15]	CS	Chile	30 (9/21)	CD = 7 (2/5) UC = 23 (7/16)	N/A	40	oral ulcerations = 1 aphthae = 4, angular ulcer = 1, ridging = 1, scar = 1, edema = 1, erythema = 2	N/A	N/A	N/A	N/A
Greuter T, 2017 [2]	RSP	Switzerland	329 (181/149)	CD = 173 (104/69) UC/NS = 156 (77/79)	N/A	12 CD: 12 UC: 11	aphtous stomatitis = 24 (5 CD, 18 UC,1 NS)	N/A	N/A	5-ASA, antibiotics, steroids, immunomodulators, anti-TNF	N/A
Mohan Kumar KP, 2016 [16]	CC	India	30 (16/14)	UC = 15 (8/7)	15 (8/7)	N/A	aphthous ulcerations = 10, lichen planus = 3, dry mouth = 11, PSV = 1, coated tongue = 4, dysgeusia = 5, halitosis = 12	N/A	periodontal index UC = 1 Ctrl = 1.4 gingival index UC = 1.2 Ctrl = 1.3 loss of attachment (mm) UC 0.4 Ctrl = 0.2	sulfapyridine; sulfasalazin	N/A
Koutsochristou V, 2015 [17]	CC	Greece	110 (50/60)	CD = 36 (18/18) UC = 19 (7/12)	55 (25/30)	12.26 ± 5.22 Ctrl: 12.21 ± 3.96	aphthae = 8, aphthae with swelling of gums or ulcers or candidiasis = 5	DMFT IBD = 5.81 (*p* < 0.001) Ctrl = 2.04 dmft IBD = 2.95 (*p* < 0.001) Ctrl = 0.91	CPITN index: IBD: score 0 = 0 (0%) score 1 = 20 (36%) score 2 = 30 (54%) score 3 = 5 (9%) Cr: score 0 = 22 (40%) score 1 = 25 (45%) score 2 = 8 (14%) score 3 = 0 (0%)	aminosalicylates, corticosteroids, anti-TNF, or immuno-modulators	N/A
Laranjeira N, 2015 [18]	CC	Portugal	171 (85/86)	CD = 65 (32/33) UC = 48 (25/23)	58 (28/30)	45.5 ± 16.9 Ctrl: 47.4 ± 16.3 CD: 41.1 ± 15.2 UC: 49.2 ± 18.4	aphthous ulcers = 10, (*p* = 0.159; gingival swelling = 1 (0.90%), angular cheilitis = 1 (0.90%), halitosis (*p* = 0.038	N/A	N/A	Corticosteroids, salicylate, immunosuppressants	Nonsmokers Ctrl = 52 CD = 52; UC = 42 Smokers Ctrl = 6 CD = 13; UC = 6
Szymanska S, 2014 [19]	CC	Sweden	225 (102/123)	CD with RS = 71 (33/38) CD without RS = 79 (40/39)	75 (29/46)	47.1 ± 24.08 Ctrl: 48.6 ± 13.4 CD with RS: 50.7 ± 13.9 CD without RS: 42 ± 14.4	dry mouth (*p* = 0.001): 29% in CD with RS and 38% in CD without RS Bad breath (*p* = 0.008) 21% in CD with RS and 33% in CD without RS Ulcers 23% in Ctrl, 23% in CD with RS and 27% in CD without RS	Ctrl = 13.1 CD with RS = 15.5 CD without RS = 11.2	N/A	N/A	Ctrl = 5 CD with RS = 17 CD without RS = 15 Former smokers Ctrl = 33 CD with RS = 25 CD without RS = 30
Zippi M, 2014 [3]	RSP	Italy	811 (437/374)	CD = 216 (131/85) UC = 595 (306/289)	N/A	32.5 ± 18.9 CD: 31.9 ± 13.1 UC: 33.1 ± 13.7	aphthous stomatitis = 3 CD e 3 UC	N/A	N/A	N/A	CD Smoker = 98. Nonsmoker = 101 Ex smokers = 17. UC Smokers = 140. Ex smokers = 131. Nonsmokers = 324
Elahi M, 2012 [20]	CC	Iran	100 (54/46)	UC: 50 (2/24)	50 (26/24)	39 ± 26.5 Ctrl: 40 ± 20 UC: 38 ± 16	Oral ulcerations = 20 (*p* = 0.028); tongue coating = 14 (*p* = 0.012); dry mouth = 30 (*p* = 0.023); halitosis = 34 (*p* = 0.001); acidic taste = 20 (*p* = 0.008), taste changes = 20 (*p* 0.001)	N/A	N/A	N/A	N/A
Habashneh RA, 2012 [21]	CC	Jordania	260 (156/104)	CD = 59 (33/26) UC = 101 (61/40)	100 (62/38)	39.4 ± 0.7	N/A	N/A	CD PPD = 1.29 0.47; CAL = 1.95 0.98. % of sites with BOP = 10.84 16.20 gingival recession = 0.53 0.55 UC PPD = 1.51 0.47; CAL = 2.36 1.13 % of sites with BOP = 10.20 14.25 gingival recession = 0.86 0.72 Ctrl PPD = 1.25 0.37, CAL = 1.70 0.89 % of sites with BOP = 4.70 8.30 gingival recession = 0.44 0.60	N/A	CD Nonsmoker = 23; Smoker = 31; ex-smoker = 5 UC nonsmoker = 55; smoker = 17; ex-smoker = 29 Ctrl nonsmoker = 44; smoker = 49; ex-smoker = 7
Jose FA, 2009 [9]	PSP/RSP	USA	1649 (893/756)	CD = 1007 UC = 471 ND = 171	N/A	11.1 ± 4.15	aphthous stomatitis = 53	N/A	N/A	N/A	N/A
Rikardsson S, 2009 [22]	CC	Sweden	2346 (32.5%/67.5%)	CD = 1598 (32%/68%)	Ctrl = 748 (33%/67%)	49.6 ± 20. 60 Ctr: 49.5 ± 13.8 CD: 49.7	oral ulcers = 32% (*p* < 0.001), halitosis = 23% (*p* < 0.001) mouth dryness = 38% (*p* < 0.001) toothaches = 21% (*p* < 0.001) mucosal lesions = 31% (*p* < 0.001)	Carious lesions = 41% (*p* < 0.001)	bleeding from gingiva = 41% (*p* < 0.001) periodontitis = 7% (*p* < 0.028)	N/A	current smokers CD = 23% Ctrl = 19% (*p* < 0.018) former smokers CD = 19% Ctrl = 15% (*p* < 0.070)
Brito F, 2008 [6]	CC	Brazil	253 (88/165)	CD: 99 (31/68) UC: 80 (33/47)	74 (24/50)	40.8 ± 22.5 Ctrl: 40.3 ± 13.2 CD: 39 ± 12.9 UC: 43.3 ± 13.2	Candidiasis = 20 (8 CD, 8 UC) Ulcerous aphtous = 3 (2 CD, 1 UC) Lichen planus = 5 (1 CD, 3 UC)	CD = 15.1 ± 7.2 (*p* = 0.018) UC = 16.4 ± 6.6 (*p* < 0.0001) Ctrl = 12.5 ± 6.8	CD PPD = 2.3 ± 1.3 mm (*p* < 0.0001; CAL = 0.9 ± 0.9 mm; % of sites with BOP = 19.6 ± 20.5 (*p* = 0.038); periodontitis = 81 (81.8%) Ctrl PPD = 1.6 ± 0.4; CAL =1.2 ± 1.0; % of sites with BOP = 24.4 ± 29.7; Periodontitis = 50 (67.6%) UC PPD = 2.3 ± 0.4 (*p* < 0.0001); CAL = 1.3 ± 1.4 (*p* = 0.004); % of sites with BOP = 21.5 21.9; Periodontitis = 72 (90%) (*p* < 0.001)	Aminosalicylates, immunomodulators, corticosteroids, antibiotics, anti TNF alpha	CD smokers = 12 (12.1%); non-smokers = 63 (63.3%); former smokers = 24 (24.3%) UC smokers = 7 (8.7%); non-smokers = 38 (47.5%); former smokers = 35 (43.8%) Ctrl smokers = 9 (12.2%); non-smokers = 57 (77%); former smokers = 8 (10.8%)
Grössner-Schreiber B, 2006 [23]	CC	Germany	121 (48/73)	CD: 46 UC: 16	Ctrl = 59 (24/35)	38.3 ± 14.3 Ctrl: 38.2 ± 10	Mucobuccal hyperplasia or oedema = 15, swelling of the gingiva = 17, ulcera = 5, Aphthae = 6, Candidiasis = 5, lichen planus = 3, leucoplachia = 2, labial rhagades = 3	DMF-S (*p* = 0.212) IBD = 54.1 ± 31.6. Cr = 46.5 ± 26.5 Dentine caries: (*p* = 0.033) IBD = 25; Ctrl = 13	BOP (*p* = 0.958) IBD = 23.4 ± 20.1; Ctrl = 20.8 ± 13.5. PPD (*p* = 0.014) IBD = 2.22 ± 0.57; Ctrl = 2.29 ± 0.33. (*p* = 0.014) CAL > 4 mm: (*p* = 0.07) IBD = 50; Ctrl = 38. CAL > 5 mm: (*p* = 0.07) IBD = 39; Ctrl = 27	Corticosteroids, immunosuppressants, aminosalicylate, anti TNF, antibiotics	IBD nonsmokers = 34; IBD smokers = 25; IBD former smokers = 3; Ctrl nonsmokers = 29; smokers = 24; former smokers = 6.
Harty S, 2005 [24]	PSP	Ireland	80	CD = 49 (25/24) UC = 22 ND = 9 oral CD = 20 (12/8) non oral CD = 28	N/A	CD: 11.95	patients with oral CD vs. nonoral CD: oral symptoms = 14 (*p* = 0.01) oral ulcerations = 11 angular stomatitis = 5 cheek swelling = 5	N/A	nonspecific gingivitis = 8	N/A	N/A
Khouri JM, 2004 [25]	RSP	Australia	6 (5/1)	CD = 4 (4/0)	N/A	6.33 CD: 6.25	lip swelling = 6 granulomatous cheilitis = 6 cobblestoning = 2	N/A	N/A	N/A	N/A
Zervou F, 2004 [26]	CC	Greece	74	CD = 15 UC = 15	44	CD: 40 ± 16 Ctrl: 43 ± 12	oral ulcerations = 3 (2 CD (*p* = 0.011); 1 UC (*p* = 0.07)) cobblestoning = 3 CD (*p* = 0.002) polypoids tags = 3 CD (*p* = 0.002) lip swelling = 4 (3 CD (*p* = 0.002); 1 UC (*p* = 0.07)) buccal swelling = 1 CD (*p* = 0.07) aphthous ulcers = 1 UC (*p* = 0.07) angular cheilitis = 9 (5 CD (*p* = 0.000); 4 UC (*p* < 0.0001)) Hairy tongue = 3 (2 CD (*p* = 0.011); 1 UC (*p* = 0.07)) buccal trauma = 9 (6 CD (*p* = 0.000); 3 UC (*p* < 0.00019))	N/A	Periodontitis = 2 CD, (*p* = 0.011) Gingivitis = 4 (3 CD (*p* = 0.002); 1 UC (*p* = 0.07)) Gingival bleeding = 4 CD (*p* < 0.0001)	mesalazine, azathioprine	N/A
Katz J, 2003 [27]	CC	Israel	96 (49/47)	CD = 34 (20/14) UC = 20 (7/13)	42 (22/20)	38.5 ± 26.9 Ctrl: 40 ± 20 CD: 33 ± 16 UC: 44 ± 18	Halitosis = 50% UC (*p* = 0.0008); 29% CD, (*p* = 0.026) dry mouth = 30% UC, (*p* = 0.04); 29% CD (*p* = 0.026) geographic tongue = 15% CD (*p* = 0.01)	N/A	N/A	N/A	N/A

**Table 2:** Newcastle-Ottawa Quality Assessment Scale scores of the individual studies.

Author, Year	Selection	Comparison	Outcome
Cagir Y, 2024 [11]	xxx	x	xx
Tabarsi NT, 2024 [12]	xxx	xx	xx
Hu S, 2022 [13]	xxx	xx	xxx
Khozeimeh F, 2020 [14]	xxx	x	xxx
Oviedo C, 2017 [15]	xxx	x	xxx
Greuter T, 2017 [2]	xxx	x	xx
Mohan Kumar KP, 2016 [16]	xxx	x	xx
Koutsochristou V, 2015 [17]	xxx	x	xxx
Laranjeira N, 2015 [18]	xxx	x	xxx
Szymanska S, 2014 [19]	xxx	xx	xx
Zippi M, 2014 [3]	xxx	xx	xxx
Elahi M, 2012 [20]	xxx	x	xxx
Habashneh RA, 2012 [21]	xxx	x	xxx
Jose FA, 2009 [9]	xxx	x	xxx
Rikardsson S, 2009 [22]	xxx	xx	xxx
Brito F, 2008 [6]	xxx	xx	xxx
Grössner-Schreiber B, 2006 [23]	xx	xx	xx
Harty S, 2005 [24]	xxx	x	xxx
Khouri JM, 2004 [25]	xxx	x	xxx
Zervou F, 2004 [26]	xxx	xx	xxx
Katz J, 2003 [27]	xxx	xx	xxx

## Data Availability

No new data were created or analyzed in this study.

## Supplementary Materials

The following supporting information can be downloaded at: https://www.mdpi.com/article/10.3390/dj14050250/s1. File S1: PRISMA 2020 Checklist.
